# Wolf phase tomography (WPT) of transparent structures using partially coherent illumination

**DOI:** 10.1038/s41377-020-00379-4

**Published:** 2020-08-19

**Authors:** Xi Chen, Mikhail E. Kandel, Chenfei Hu, Young Jae Lee, Gabriel Popescu

**Affiliations:** Quantitative Light Imaging Laboratory, Beckman Institute for Advanced Science and Technology, Department of Electrical and Computer Engineering, University of Illinois at Urbana-Champaign, Urbana, IL 61801 USA

**Keywords:** Phase-contrast microscopy, Imaging and sensing, Phase-contrast microscopy, Imaging and sensing

## Abstract

In 1969, Emil Wolf proposed diffraction tomography using coherent holographic imaging to extract 3D information from transparent, inhomogeneous objects. In the same era, the Wolf equations were first used to describe the propagation correlations associated with partially coherent fields. Combining these two concepts, we present Wolf phase tomography (WPT), which is a method for performing diffraction tomography using partially coherent fields. WPT reconstruction works directly in the space–time domain, without the need for Fourier transformation, and decouples the refractive index (RI) distribution from the thickness of the sample. We demonstrate the WPT principle using the data acquired by a quantitative-phase-imaging method that upgrades an existing phase-contrast microscope by introducing controlled phase shifts between the incident and scattered fields. The illumination field in WPT is partially spatially coherent (emerging from a ring-shaped pupil function) and of low temporal coherence (white light), and as such, it is well suited for the Wolf equations. From three intensity measurements corresponding to different phase-contrast frames, the 3D RI distribution is obtained immediately by computing the Laplacian and second time derivative of the measured complex correlation function. We validate WPT with measurements of standard samples (microbeads), spermatozoa, and live neural cultures. The high throughput and simplicity of this method enables the study of 3D, dynamic events in living cells across the entire multiwell plate, with an RI sensitivity on the order of 10^−5^.

## Refractive Index Analysis: Wolf Phase Tomography

A scheme for determining the three-dimensional refractive index distribution of transparent structures could prove useful for monitoring morphological changes in living cells. Xi Chen and coworkers from the University of Illinois in the USA say that their technique, called Wolf Phase Tomography (WPT), has the advantages of simple sample preparation, high throughput and also high sensitivity (refractive index variations on the order of 10^-5^ can be detected). The team analyzed several samples using WPT including a suspension of 2-µm polystyrene microspheres in immersion oil, sperm cells and neurons. The approach makes use of an adapted phase-contrast microscope and partially coherent illumination (a ring-shaped white light source). Refractive index mapping of biological cells and tissue is useful for the study of cancer and cellular transport and mitosis.

## Introduction

The refractive index (RI) is a fundamental physical property that determines how light interacts with a medium in terms of scattering, governed by its real part, and absorption, through its imaginary part^1–5^. In biological applications, the RI distribution correlates strongly with cellular properties, such as dry mass and chemical concentrations^6–9^. Tissue RI can also act as an intrinsic marker for cancer diagnosis^10,11^. Nanoscale morphological changes in cells and tissues can be revealed by RI maps^12,13^. For example, it has been shown that cancer tissue exhibits higher RI variances than normal tissue^10,11^. The RI can also be used to study biological dynamics, including cellular transport and mitosis^14,15^, and can be used for phenotypic screening and cellular monitoring^16,17^. To obtain the RI distribution of cells and tissues from the measured field properties in different imaging modalities, one must go beyond the typical quantities measured in phase imaging and solve an inverse scattering problem. A condition for this problem to yield unique solutions is to measure the full information about the scattered field, meaning both the amplitude and phase. Interferometric microscopy provides a method for phase retrieval in weakly scattered samples such as cells and tissues^18–20^.

Quantitative-phase imaging (QPI) has emerged as a growing field focused on extracting the phase distributions of an imaging field and exploiting that information for biomedical applications^18,21–27^. White-light-based methods, such as spatial light interference microscopy (SLIM)^28^, gradient light interferometric microscopy^29^, and (white-light) diffraction-phase microscopy^30^, can render phase images of live cells without the speckle noise typically associated with coherent illumination^21^. As a result, the spatial sensitivity to pathlength changes is very high. The optical pathlength measurement depends on both the RI and the thickness of the sample^31^. To estimate the 2D (axially averaged) RI from the optical pathlength, the thickness distribution of the structures needs to be known or decoupled from the optical pathlength^32,33^. However, the accuracy is low due to the geometrical optics approximation, and the results provide only a 2D map of the longitudinally averaged RI.

For inferring the 3D RI distribution from QPI data, several approaches have been proposed based on solving the deterministic wave equations^34,35^. One of them is the filtered back-projection algorithm, which uses the Fourier diffraction theorem and the first-order Born or Rytov approximation^25^. It connects the object function with the Fourier transform of the projection. The reconstruction of the RI distribution is obtained by combining the frequency bands with respect to different angles^36^. One can achieve this by rotating the illumination angles or measuring a set of image fields at successive points across a cell with focused beam illumination, also known as synthetic aperture tomography-phase microscopy^37^. However, the Fourier diffraction theorem^34^ assumes plane-wave illumination, and is only an approximation for partially coherent fields. To obtain a more accurate solution to the inverse problem, the coherence properties must be taken into account. White-light diffraction tomography (WDT) uses the temporal correlation and instrument response to perform deconvolution on the complex field data to extract the 3D scattering potential^38^. However, WDT requires a priori knowledge of the instrument impulse response (or transfer function), which is often limited. At the same time, deconvolution operations are usually time-consuming and sensitive to noise.

The transport of the intensity equation can connect the RI to the intensity of bright-field images^39,40^. However, this method is only applicable under a paraxial approximation (see, e.g., Section 12.1 in ref. ^18^). Integrated microchips have been used to measure RI information, combining an external cavity laser, microlenses, and microfluidic channels into a monolithic device^41,42^. Such a device can determine the average RI of a single live cell in real time, but cannot render the RI distribution. 3D RI distributions are usually constructed through axial scanning (z scanning)^29,38,43^ or projections from different angles (computed tomography)^25,26,44^. To increase the axial resolution in 3D reconstruction, efforts have been devoted toward alleviating the incomplete frequency coverage of imaging systems, or “the missing cone problem”^45^. Illumination angle scanning and rotation of the sample can help fill in a missing cone region. Cells can be rotated by optical tweezers or dielectrophoretic forces in microfluidics^46–49^. However, these methods involve more complicated procedures.

In this paper, we propose a fast 3D RI construction method based on the Wolf equations for propagating correlations of partially coherent light^50,51^. This approach, referred to as Wolf phase tomography (WPT), involves minimal computational steps, and renders high-resolution RI tomograms without time-consuming deconvolution operations. WPT decouples the RI distribution from the thickness of the sample in the space–time domain directly, without the need for Fourier transformation. We demonstrate that from three independent intensity measurements corresponding to each phase shift, the RI distribution can be reconstructed directly from the Laplacian and second time derivative of the complex correlation functions. We demonstrate WPT with standard polystyrene beads, fixed spermatozoa, and dynamic live-cell imaging over many hours. Interestingly, we find that WPT is able to extract intrinsic RI changes in live cells with a sensitivity on the order of 10^−5^, which can indicate cell viability in screening applications.

## Results

### WPT principle

**Fig. 1:**
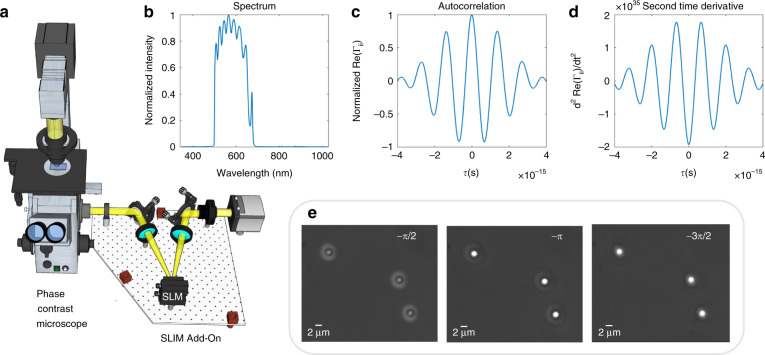
Optical setup and working principle of Wolf phase tomography (WPT). **a** WPT optical setup. The spatial light interference microscopy (SLIM) add-on module is mounted to the output camera port of a phase-contrast microscope. This module shifts the phase between the incident and scattered field with *π*/2 increments using the SLM. The interference patterns are recorded by the camera. **b** Spectrum, **c** autocorrelation, and **d** second-order derivative of the autocorrelation of the halogen source measured by the spectrometer. **e** Three phase-shifted frames of 2-μm polystyrene beads are acquired using the SLIM module (×63/1.4 NA)

WPT relies on a commercial phase-contrast microscope upgraded with a spatial light modulator (SLM) conjugated to the pupil plane. In our implementation, this hardware is provided by a SLIM module (SLIM Pro, Phi Optics), as shown in Fig. 1a. Figure 1b–d shows the temporal spectrum and autocorrelation properties of the illumination (white-light) field. In addition to the *π*/2 phase shift between the incident and scattered fields introduced by the objective-phase ring, the SLIM module provides further phase shifts with *π*/2 increments. At the camera plane, we record three intensity images, corresponding to each phase shift, as illustrated in Fig. 1e, namely

1 $$I_d({\mathbf{r}}) = I_i({\mathbf{r}}) + I_S({\mathbf{r}}) + 2\Re [{\Gamma} _{is}\left( {\left\langle \omega \right\rangle \tau _d + {\Delta} \phi ({\mathbf{r}})} \right)]$$

where $\left\langle \omega \right\rangle \tau _d = - d\pi /2$*, d* = 1, 2, 3, $\left\langle \omega \right\rangle$ is the central frequency of the incident field, $\Re$ stands for the real part, ${\Delta} \phi$ is the phase difference between the incident field *U*_*i*_ and scattered field *U*_*S*_, and ${\Gamma} _{pq}({\mathbf{r}}_1,{\mathbf{r}}_2,\tau ) = \langle U_p^ \ast ({\mathbf{r}}_1,t)U_q({\mathbf{r}}_2,t + \tau )\rangle _t$, $p,q = \{ i,s\} .$ From these three frames, we solve for $\Re \left[ {{\Gamma} _{is}(\left\langle \omega \right\rangle \tau _d + {\Delta} \phi ({\mathbf{r}}))} \right]$. Based on partially coherent light propagation, governed by the Wolf equations^51^, the RI of the object can be obtained by (see the full derivation in Supplementary Note 1)

2 $$n({\mathbf{r}}) = \sqrt {\frac{{m({\mathbf{r}}) - n_0^2\left[ {1 - g({\mathbf{r}})} \right]}}{{1 + g({\mathbf{r}})}}}$$

In Eq. (2), the functions *m* and *g* are defined as

3a $$m({\mathbf{r}}) = \left. {\frac{{c^2\left( {\nabla ^2\Re [{\Gamma} _{is}({\mathbf{r}},{\mathbf{r}},\tau )] + \zeta ({\mathbf{r}})} \right)}}{{\frac{{\partial ^2\Re [{\Gamma} _{is}({\mathbf{r}},{\mathbf{r}},\tau )]}}{{\partial \tau ^2}}}}} \right|_{\tau = - \pi /\left\langle \omega \right\rangle }$$

3b $$\zeta ({\mathbf{r}}) = - 2\Re \mathop {\int}\limits_0^\infty {\langle \nabla U_i^ \ast ({\mathbf{r}},\omega ) \cdot \nabla U_s({\mathbf{r}},\omega )\rangle e^{i\omega \pi /\left\langle \omega \right\rangle }d\omega }$$

3c $$g({\mathbf{r}}) = \left. {\frac{{\frac{{\partial ^2\Re [{\Gamma} _{ii}({\mathbf{r}},{\mathbf{r}},\tau )]}}{{\partial \tau ^2}}}}{{\frac{{\partial ^2\Re [{\Gamma} _{is}({\mathbf{r}},{\mathbf{r}},\tau )]}}{{\partial \tau ^2}}}}} \right|_{\tau = - \pi /\left\langle \omega \right\rangle }$$

where **r** = (*x*, *y*, *z*) is the spatial coordinate, *n*_0_ is the RI of the background media, and *c* is the speed of light in vacuum. The detailed steps for calculating the terms in Eqs. (3a)–(3c) are given in Supplementary Note 1. The term in Eq. (3b) does not substantially contribute to the final RI and can be omitted for faster construction (see the discussion in Supplementary Note 2). Figure 1b describes the normalized spectrum of a halogen source measured by the spectrometer (ocean optics). The real part of the normalized autocorrelation $\Re [{\Gamma} _{ii}({\mathbf{r}},{\mathbf{r}},\omega _0\tau )]$ is obtained by taking the Fourier transform of the spectrum (see Fig. 1c). To retrieve the temporal correlation function quantitatively, we normalized the Γ_*ii*_(**r**,**r**,0) value from the spectrometer data to the background intensity from the camera, and corrected it with the spectrally dependent quantum efficiency of the camera. Thus, we ensured that the autocorrelations Γ_*ii*_(**r**,**r**,0) measured by the two different devices have the same value. The second-order time derivative of Γ_*ii*_(**r**,**r**,*τ*) is depicted in Fig. 1d. The Laplacian in Eq. (3a) is calculated using three images with a first-order finite-difference approximation. The z component of the Laplacian was computed using three axially distributed frames separated by a distance that matches the *x*–*y* pixel sampling, and is much smaller than the diffraction spot. For example, for a ×40/0.75 NA objective, this distance is 0.14 μm, while the diffraction-limited resolution is 0.4 μm. The second-order derivatives in Eqs. (3a) and (3c) are calculated in MATLAB using three phase-shifted frames. Smaller phase shifts would give more accurate derivatives. However, the contrast between different frames would greatly decrease; thus, the signal-to-noise ratio would decrease as well, resulting in a lower accuracy for the derivative. Therefore, to increase the signal-to-noise ratio and accuracy, we keep the phase increment at *π*/2. This algorithm requires 40 ms to reconstruct the RI map at one z position with a 3-megapixel field of view (MATLAB, i7-8650U CPU).

### WPT on standard samples

**Fig. 2:**
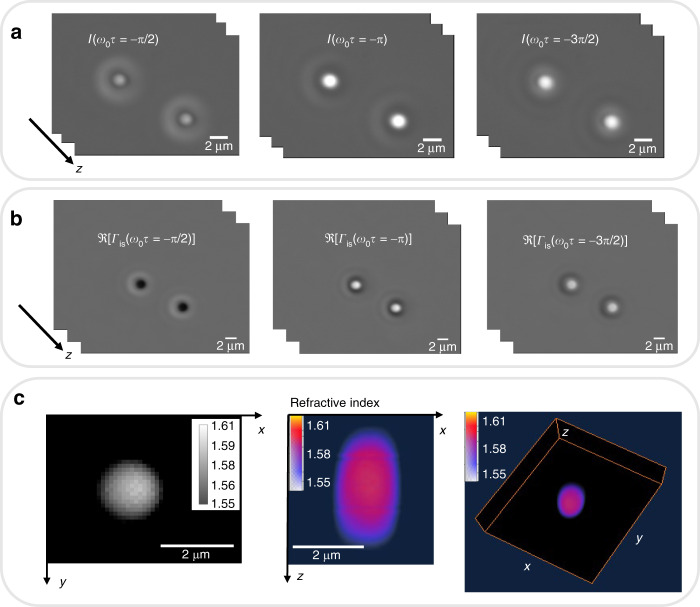
Wolf phase tomography (WPT) on standard samples. **a** Three phase-shifted frames of 2-μm polystyrene beads suspended in oil are imaged by spatial light interference microscopy (SLIM) using a ×63/1.4 NA objective. **b** The real part of the correlation function at three different time delays is obtained by solving Eq. (1). **c** 3D refractive index (RI) tomogram of the 2-μm polystyrene bead

To validate the capability of WPT in extracting the RI distribution, we imaged 2-μm polystyrene microsphere (Polysciences Inc.) z stacks with an RI value of 1.59 at the central wavelength. The beads are suspended in immersion oil (Zeiss) with an RI value of 1.518. Figure 2a shows the three frames corresponding to the different phase shifts of the polystyrene beads. For these experiments, we use a ×63/1.4 NA objective. The real parts of the correlation function Γ_*is*_ at different time-lapse values are illustrated in Fig. 2b. The RI distribution of the microspheres for each z slice is reconstructed via Eq. (2). The 3D rendering of the RI distribution described in Fig. 2c was obtained in AMIRA (Thermo Fisher Scientific). The reconstructed RI value of the microspheres agrees well with the expected value of 1.59 at the central wavelength. The halo artifacts associated with phase-contrast and SLIM images were removed from the final RI maps using our previously reported algorithm^52^.

### WPT of sperm cells

**Fig. 3:**
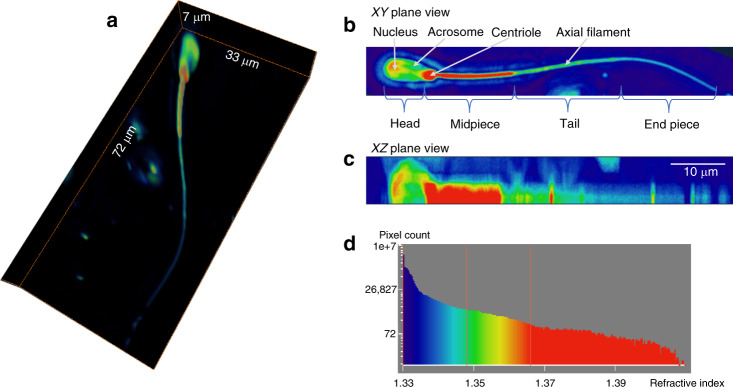
Wolf phase tomography (WPT) of sperm cells. **a** 3D refractive index (RI) tomogram of a spermatozoon (×40/0.75 NA objective). **b** *xy*-plane projection view. The nucleus, acrosome, centriole, and axial filament of the sperm cell are indicated by white arrows. **c** *xz*-plane projection view. **d** Histogram of the RI of the sperm cell

A 3D rendering of a bovine sperm cell is displayed in Fig. 3a (Supplementary Video 1). In the sperm head, the acrosome and the nucleus can be identified with RI values between 1.35 and 1.37. The centriole and mitochondria-rich midpiece of the sperm cell yield high RI values (Fig. 3b). The tail of the sperm has an RI value of 1.35, and the axial filament inside the tail, with a slightly higher RI value of 1.36, can be recognized. The end piece of the sperm has the lowest RI value, ~1.34.

### WPT of neurons

**Fig. 4:**
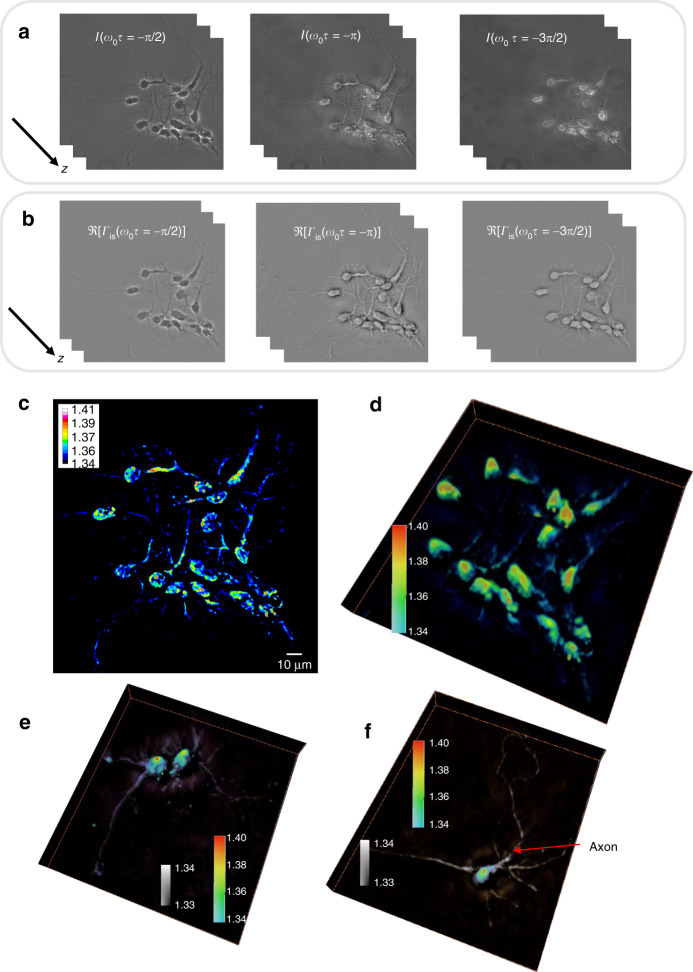
Wolf phase tomography (WPT) of neurons. **a** Three phase-shifted frames of hippocampal neurons (×40/0.75 NA objective). **b** The real part of the correlation function at three different time lags is solved with Eq. (1). **c** Refractive index (RI) map of hippocampal neurons. **d**–**f** 3D rendering of RI tomograms of hippocampal neurons. **e**, **f** Two colormaps are used as indicated to enhance the dendrites and axons. The axon is indicated with a red arrow

Applying the WPT principle, the three frames of hippocampal neurons and their correlation functions are depicted in Fig. 4a and b. The reconstructed RI distribution and 3D rendering of the neurons (Supplementary Video 2) are displayed in Fig. 4c and d. The more detailed structures of individual hippocampal neurons (Supplementary Videos 3 and 4) are illustrated in Fig. 4e and f. The rendering in this case used two colormaps, as shown in Fig. 4e and f. The neuron dendrites have an RI value of ~1.34, while the cell body ranges from 1.35 to 1.38, with a nucleolus of 1.39–1.4. The axon can be recognized in Fig. 4f, as the morphology shows a longer and thinner filamentous structure.

### Dynamic WPT of live cells

**Fig. 5:**
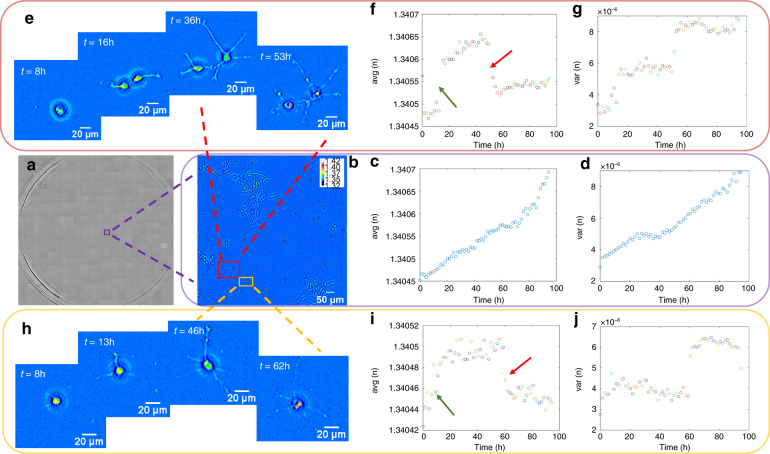
Dynamic Wolf phase tomography (WPT) of live cells across multiwell plates. **a** The refractive index (RI) map across a whole well of living hippocampal neurons (×10/0.3 NA objective) is composed of 20 × 21 mosaic tiles, each 214 × 204 µm^2^ in area. **b** Enlarged RI map of the purple box in (**a**) with the average (**c**) and variance (**d**) of the RI versus time. **e** Enlarged RI map of the area in the red box in (**b**) with the average (**f**) and variance (**g**) of the RI. The green arrow indicates the increase in the RI when the two neurons separated and their dendrites appeared, and the red arrow shows the decrease in RI when the two neurons died. **h** Enlarged RI map of the area in the yellow box in (**b**) with the average (**i**) and variance (**j**) of the RI. The green arrow indicates the change in the RI when the dendrites appeared, and the red arrow indicates the decrease in RI when the neuron died

Due to its high throughput, low phototoxicity, absence of photobleaching, and easy sample preparation, WPT is capable of studying real-time volumetric biological events in living cells. We imaged the growth and proliferation of hippocampal neurons over the course of several days in six-well plates typical in phenotypic screening applications. The RI distribution of the whole well of neurons is displayed in Fig. 5a (Supplementary Video 5). One tile zoom-in of the whole well and its distribution of RI is shown in Fig. 5b (Supplementary Video 6). Figure 5c describes the averages of the RI values within this tile versus time. The average RI values increase with time due to neuron growth. Figure 5c illustrates the average RI of the whole tile, including the neurons and background. As the neurons grow, more pixels in the region of interest appear with higher RIs; thus, the average RI becomes larger. Another point worth mentioning is that the range of the *y* axis in Fig. 5c is from 1.34045 to 1.34070. Thus, due to the averaging over the large field of view, the change in the RI value detected by our system is at the fifth decimal place, indicating the high sensitivity of WPT. Figure 5d shows that the variance of the RI for this tile increases with time as well^53^. Note that the range of RI variance values is on the order of 10^−6^, which is detectable due to the sensitivity conferred by the common-path stability and lack of speckles in SLIM.

Figure 5e is the enlarged image of the area in the red box in Fig. 5b containing two neurons. The neurons spread out into two regions at ~*t* = 16 h, continued growing until ~*t* = 53 h, and then died. We can see that both the average and variance of the RI show three different stages (Fig. 5f, g). One significant change in the average and variance of the RI appeared when the two neurons separated (red arrows). Another change is visible when the two neurons died (green arrows). The death event was accompanied by a decrease in the mean RI, likely due to the membrane permeability, which allowed for water influx.

**Fig. 6:**
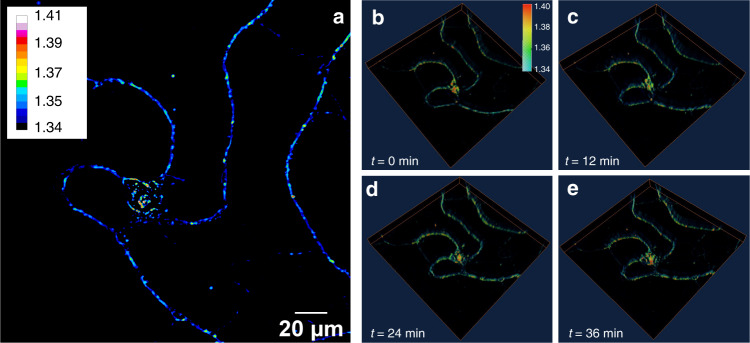
Time-lapse Wolf phase tomography (WPT) of live neurons. **a** Refractive index (RI) map of a live hippocampal neuron imaged with a ×40/0.75 NA objective. **b**–**e** 3D RI tomograms of the hippocampal neuron 12-min apart

Figure 5h is a magnified image of the area in the yellow box in Fig. 5b containing one neuron. The neuron dendrites started to appear at ~*t* = 13 h, resulting in a jump in the average RI (Fig. 5i). The neuron continued growing until ~*t* = 62 h and then died, leading to a decrease in the average RI. Some oscillations in the variance (Fig. 5j) of the RI appeared before the neuron died and exhibited a clear change after the neuron died. Figure 6 demonstrates the capability of WPT for 3D real-time live-cell imaging. The changes in the morphology of the neuron can be recognized at different time frames.

## Discussion

In summary, we proposed a new high-throughput RI tomography method, WPT, based on the correlation propagation of partially coherent light. We demonstrated the capability of WPT with tomographic reconstructions of standard polystyrene beads, spermatozoa, and hippocampal neurons. Our method builds on the coherence theory pioneered by Emil Wolf by combining the Wolf equations and diffraction tomography to perform the reconstruction directly in the space–time domain, without the need for Fourier transformation. WPT decouples the RI distribution from the thickness of the object by calculating the Laplacian and the second-order time derivative of the complex correlation functions. High-resolution tomograms of RI distributions are acquired using a z stack of three phase-shifted intensity frames. As a result, the tomographic reconstruction is very fast, requiring only 40 ms per z slice. In our implementation, the total acquisition time is 180 ms per z slice. With more advanced SLMs, z-scanning stages, and cameras, ~40 ms of the total acquisition time can be achieved per z slice. WPT has a high RI sensitivity, on the order of 10^−5^, which is useful as an intrinsic marker for live-cell monitoring. We illustrated this ability by imaging dynamic live cells over many hours. As a label-free method, WPT is nondestructive and is not limited by the photobleaching and phototoxicity commonly associated with fluorescence microscopy.

In a larger context, WPT highlights the advantage of partially coherent illumination, phase shifting, and phase-contrast geometry. The white-light illumination and common-path interferometry allow for speckle-free and nanometer-path-length stability. As phase shifting is performed in the pupil plane, in the time domain, our reconstruction preserves the diffraction-limited resolution of the microscope without introducing coherent artifacts (such as residual fringes)^54^.

Although the mathematical model derived in WPT does not use approximation, the ability to access the information of the correlation functions is limited experimentally to weakly scattering samples. It is also worth mentioning that since we use white light with a very short coherence length (~1–2 µm), long optical paths are cut off via coherence gating. This implies that within the optical section, scattering is characterized well by the Born approximation, even though the entire specimen might produce multiple scattering. However, WPT is still limited by the “missing cone problem” due to its finite numerical aperture. Therefore, WPT can also adopt other methods, such as cell rotation and illumination angle scanning, to achieve better axial resolution and depth sectioning. As an alternative, new advances in deep learning appear promising in addressing this issue by frequency extrapolation^55^. We envision that WPT will find important applications in material and life sciences, such as studying adherent cell growth with the segmentation of a nucleus compared to the whole cell, cell classification based on RI, histopathology for cancer diagnosis based on RI^10^, and 3D tracking of collagen fibers.

## Materials and methods

### SLIM add-on module

The SLIM add-on module is mounted to the output camera port of a commercial phase-contrast microscope. The module contains an SLM (Meadowlark, XY Series) and a camera (Hamamatsu, ORCA-Flash 4.0 V2). Measurements were conducted using an inverted microscope (Zeiss, Axio Observer Z1) with a halogen light source (Zeiss, HAL 100). Cells were imaged with an incubation system under ×63/1.4 NA, ×40/0.75 NA, and 10×0.3 NA objectives with matching phase-contrast illumination. The sampling was uniform in the *x*, *y*, and *z* directions. For example, for a ×40/0.75 NA objective, the distance is 0.14 μm, which is smaller than the diffraction-limited resolution of 0.4 μm.

The 40-ms reconstruction per frame is faster than the SLIM image acquisition rate of 180 ms, which requires 30 ms for SLM stabilization (Meadowlark XY Series), 10 ms for exposure (Hamamatsu, V2 Orca Flash), and 60 ms for z scanning. We expect the technique to be able to be implemented on faster hardware without modification.

### Sample preparation

The hippocampal neurons were prepared as follows: primary neurons were harvested from dissected hippocampi of Sprague–Dawley rat embryos. The hippocampi were dissociated with the enzyme to obtain hippocampal neurons. The hippocampal neurons were then plated onto a six-well plate that was precoated with poly-D-lysine (0.1 mg/ml, Sigma-Aldrich). The hippocampal neurons were initially incubated with plating medium containing 86.55% MEM Eagle’s with Earle’s BSS (Lonza), 10% fetal bovine serum (refiltered, heat-inactivated, Thermo Fisher), 0.45% of 20% (wt./vol.) glucose, 1 × 100 mM sodium pyruvate (100×, Sigma-Aldrich), 1 × 200 mM glutamine (100×, Sigma-Aldrich), and 1× penicillin/streptomycin (100×, Sigma-Aldrich) to facilitate the attachment of neurons (300 cells/mm^2^). After 3 h of incubation in an incubator (37 °C and 5% CO_2_), the plating medium was aspirated and replaced with maintenance medium containing Neurobasal^TM^ growth medium supplemented with B-27 (Thermo Fisher), 1% 200 mM glutamine (Thermo Fisher), and 1% penicillin/streptomycin (Thermo Fisher) at 37 °C in the presence of 5% CO_2_. The hippocampal neurons in Fig. 5 were grown for 2 days in vitro, and dynamic images were taken for 4 days. The hippocampal neurons in Fig. 6 were grown for 14 days in vitro, and snapshots were taken every 12 min. The hippocampal neurons in Fig. 4 were fixed. The sperm cells in Fig. 3 were fixed in 10% paraformaldehyde.

## Supplementary information

Supplemental text

Supplemental video 1

Supplemental video 2

Supplemental video 3

Supplemental video 4

Supplemental video 5

Supplemental video 6

## Data Availability

The data that support the findings in this study are available from the corresponding author upon reasonable request.

## Code availability

The code and computer algorithms that support the findings of this study are available from the corresponding author upon reasonable request.
