# Supplementary material for: Wolf phase tomography (WPT) of transparent structures using partially coherent illumination
Source: Light Sci Appl. 2020 Aug 19;9:142. doi: 10.1038/s41377-020-00379-4 (PMC7438521; doi:10.1038/s41377-020-00379-4)
Supplement: Supplementary file 1 — Supplemental text [file 41377_2020_379_MOESM1_ESM.docx]

Supplementary information for

**Wolf phase tomography (WPT) of transparent structures using partially coherent illumination**

*Xi Chen, Mikhail E. Kandel, Chenfei Hu, Young Jae Lee, and Gabriel Popescu*

**Supplementary Note 1: WPT reconstruction model**

Let us consider the stochastic scalar fields generated by a partially coherent source propagating in an object with refractive index distribution $n(\mathbf{r})$. We define the scattering potential to be $\chi(\mathbf{r})=n^{2}(\mathbf{r})-n_{0}^{2}$, where $n_{0}^{2}$ is the refractive index (RI) of the background. Each realization of the stochastic incident field satisfies the Helmholtz equation [1]

$$\nabla_{2}^{2}U_{i}(\mathbf{r}_{2},\omega)+\beta_{0}^{2}n_{0}^{2}U_{i}(\mathbf{r}_{2},\omega)=0,$$

where $\beta_{0}=\omega_{0}/c$ is the wavenumber in vacuum, the is the Laplacian operator taken with respect to the point $\mathbf{r}_{2}$. While every realization of the total field $U(\mathbf{r})=U_{i}(\mathbf{r})+U_{s}(\mathbf{r})$ obeys the inhomogeneous Helmholtz equation

$$\nabla_{2}^{2}U(\mathbf{r}_{2},\omega)+\beta_{0}^{2}n^{2}(\mathbf{r}_{2})U(\mathbf{r}_{2},\omega)=0.$$

Subtracting Eq. from Eq. , we arrive at

$$\nabla_{2}^{2}U_{s}(\mathbf{r}_{2},\omega)+\beta_{0}^{2}\chi(\mathbf{r}_{2})U_{i}(\mathbf{r}_{2},\omega)+\beta_{0}^{2}n^{2}(\mathbf{r}_{2})U_{s}(\mathbf{r}_{2},\omega)=0.$$

Multiplying both sides of Eq. by $U_{i}^{*}(\mathbf{r}_{1},\omega)$, we have

$$\nabla_{2}^{2}[U_{i}^{*}(\mathbf{r}_{1},\omega)U_{s}(\mathbf{r}_{2},\omega)]+\beta_{0}^{2}\chi(\mathbf{r}_{2})U_{i}^{*}(\mathbf{r}_{1},\omega)U_{i}(\mathbf{r}_{2},\omega)$$

$$+\beta_{0}^{2}n^{2}(\mathbf{r}_{2})U_{i}^{*}(\mathbf{r}_{1},\omega)U_{s}(\mathbf{r}_{2},\omega)=0.$$

Note that we may place $U_{i}^{*}(\mathbf{r}_{1},\omega)$ under the Laplacian operator because that it was taken with respect to $\mathbf{r}_{2}$. Taking the ensemble average of all the realizations will return the correlation propagation function

$$\nabla_{2}^{2}W_{is}(\mathbf{r}_{1},\mathbf{r}_{2},\omega)+\beta_{0}^{2}\chi(\mathbf{r}_{2})W_{ii}(\mathbf{r}_{1},\mathbf{r}_{2},\omega)+\beta_{0}^{2}n^{2}(\mathbf{r}_{2})W_{is}(\mathbf{r}_{1},\mathbf{r}_{2},\omega)=0,$$

where the spectral density function is defined as

$$W_{pq}(\mathbf{r}_{1},\mathbf{r}_{2},\omega)=\langle U_{p}^{*}(\mathbf{r}_{1},\omega)U_{q}(\mathbf{r}_{2},\omega)\rangle, p,q=\{i,s\}.$$

Be aware that Eq. does not hold anymore if $\mathbf{r}_{1}=\mathbf{r}_{2}$ due to the fact that the Laplacian does not only take on one position. Instead, two more terms will appear in the expression

$$\nabla^{2}W_{is}(\mathbf{r},\mathbf{r},\omega)-2\langle\nabla U_{i}^{*}(\mathbf{r},\omega)\cdot\nabla U_{s}(\mathbf{r},\omega)\rangle+\beta_{0}^{2}n_{0}^{2}W_{is}(\mathbf{r},\mathbf{r},\omega)$$

$$+\beta_{0}^{2}\chi(\mathbf{r})W_{ii}(\mathbf{r},\mathbf{r},\omega)+\beta_{0}^{2}n^{2}(\mathbf{r})W_{is}(\mathbf{r},\mathbf{r},\omega)=0.$$

Next, we note that mutual coherence function relates to the cross-spectral function via the Fourier transform

$$\Gamma(\mathbf{r},\mathbf{r},\tau)=\int_{0}^{\infty} W(\mathbf{r},\mathbf{r},\omega)e^{-i\omega\tau}d\omega.$$

Taking the Fourier transformation on both sides of Eq. , it becomes

$$\begin{matrix} \nabla^{2}\Gamma_{is}(\mathbf{r},\mathbf{r},\tau)-2\int_{0}^{\infty} \langle\nabla U_{i}^{*}(\mathbf{r},\omega)\cdot\nabla U_{s}(\mathbf{r},\omega)\rangle e^{-i\omega\tau}d\omega\\ \begin{matrix} & \end{matrix}-\frac{[n_{0}^{2}+n^{2}(\mathbf{r})]}{c^{2}}\frac{\partial^{2}\Gamma_{is}(\mathbf{r},\mathbf{r},\tau)}{\partial\tau^{2}}-\frac{\chi(\mathbf{r})}{c^{2}}\frac{\partial^{2}\Gamma_{ii}(\mathbf{r},\mathbf{r},\tau)}{\partial\tau^{2}}=0. \end{matrix}$$

Now if we express the complex mutual coherence function as $\Gamma_{is}(\mathbf{r},\mathbf{r},\tau)=\Re[\Gamma_{is}(\mathbf{r},\mathbf{r},\tau)]+i\Im[\Gamma_{is}(\mathbf{r},\mathbf{r},\tau)]$, where $\Re$ and $\Im$ denote the real and imaginary parts. Taking the real part and evaluating at $\tau=-\pi/\left\langle\omega\right\rangle$, where $\left\langle\omega\right\rangle$is the central frequency of the illumination source, Eq. becomes

$$\begin{matrix} \nabla^{2}\Re[\Gamma_{is}(\mathbf{r},\mathbf{r},-\pi/\left\langle\omega\right\rangle)]-2\Re\int_{0}^{\infty} \langle\nabla U_{i}^{*}(\mathbf{r},\omega)\cdot\nabla U_{s}(\mathbf{r},\omega)\rangle e^{i\omega\pi/\left\langle\omega\right\rangle}d\omega\\ -\frac{[n_{0}^{2}+n^{2}(\mathbf{r})]}{c^{2}}\left.\frac{\partial^{2}\Re[\Gamma_{is}(\mathbf{r},\mathbf{r},\tau)]}{\partial\tau^{2}}\right|_{\tau=-\pi/\left\langle\omega\right\rangle}-\frac{\chi(\mathbf{r})}{c^{2}}\left.\frac{\partial^{2}\Re[\Gamma_{ii}(\mathbf{r},\mathbf{r},\tau)]}{\partial\tau^{2}}\right|_{\tau=-\pi/\left\langle\omega\right\rangle}=0. \end{matrix}$$

Therefore, the RI distribution of the object can be reconstructed as

$$n(\mathbf{r})=\sqrt{\frac{m-n_{0}^{2}(1-g)}{1+g}},$$

where

$m(\mathbf{r})=\left.\frac{c^{2}\left(\nabla^{2}\Re[\Gamma_{is}(\mathbf{r},\mathbf{r},\tau)]+\zeta(\mathbf{r})\right)}{\frac{\partial^{2}\Re[\Gamma_{is}(\mathbf{r},\mathbf{r},\tau)]}{\partial\tau^{2}}}\right|_{\tau=-\pi/\left\langle\omega\right\rangle},$ (11a)

$\zeta(\mathbf{r})=-2\Re\int_{0}^{\infty} \langle\nabla U_{i}^{*}(\mathbf{r},\omega)\cdot\nabla U_{s}(\mathbf{r},\omega)\rangle e^{i\omega\pi/\left\langle\omega\right\rangle}d\omega,$ (11b)

$g(\mathbf{r})=\left.\frac{\frac{\partial^{2}\Re[\Gamma_{ii}(\mathbf{r},\mathbf{r},\tau)]}{\partial\tau^{2}}}{\frac{\partial^{2}\Re[\Gamma_{is}(\mathbf{r},\mathbf{r},\tau)]}{\partial\tau^{2}}}\right|_{\tau=-\pi/\left\langle\omega\right\rangle},$ (11c)

Shown in Supplemental Note 2, $\zeta(\mathbf{r})$ in Eq. (11b) has a negligible contribution to the final RI distribution. The real part of the mutual correlation function is readily obtained with the SLIM add-on interferometer (Phi Optics Inc). Instead of recovering the phase information, as in typical processing, we use three phase-contrast frames to recover the refractive index as follows. The three frames recorded have the form

where $\left\langle\omega\right\rangle\tau_{d}=-d\pi/2$*,* $d=1,2,3$, is the phase difference between the incident field, $U_{i}$, and scattered field, $U_{s}$, and$\Gamma_{pq}(\mathbf{r}_{1},\mathbf{r}_{2},\tau)=\langle U_{p}^{*}(\mathbf{r}_{1},t)U_{q}(\mathbf{r}_{2},t+\tau)\rangle_{t}$, $p,q=\{i,s\}.$Writing these three terms explicitly

Notice that the magnitude of $\left|\Gamma_{is}\right|$varies slowly within the three shifts that we choose, we assume that $\left|\Gamma_{is}\right|$ does not change for each phase shift. Thus, we have

Therefore, from these three frames, we are able to solve for . In the experiment, we used partially coherent annular source, which can be characterized by the partially coherent Schell model, meaning the degree of coherence is only a function of the difference between two different positions on the source [2]. The cross-spectral function of Schell model has the form

$$W_{ii}(\mathbf{r}_{1},\mathbf{r}_{2},\omega)\text{ }=\iint p(\mathbf{v})a^{*}(\mathbf{r}_{1},\omega)e^{2\pi i\mathbf{r}_{1}\cdot\mathbf{v}}a(\mathbf{r}_{2},\omega)e^{-2\pi i\mathbf{r}_{2}\cdot\mathbf{v}}d\mathbf{v}$$

$$=a^{*}(\mathbf{r}_{1},\omega)a(\mathbf{r}_{2},\omega)\iint p(\mathbf{v})e^{-2\pi i\mathbf{r}_{d}\cdot\mathbf{v}}d\mathbf{v},$$

where $\mathbf{r}_{d}\equiv\mathbf{r}_{2}-\mathbf{r}_{1}$, $a(\mathbf{r},\omega)$ is the amplitude of the incident field at position $\mathbf{r}$, $p(\mathbf{v})$ is the spectral density of the incident field in the source plane. Thus$p(\mathbf{v})$is constant when $R_{1}\leq|\mathbf{v}|\leq R_{2}$ and zero otherwise, where $R_{1}$ and $R_{2}$ are the inner and outer radii of the phase ring in the condenser. After the integration over the source plane, Eq. becomes

$$W_{ii}(\mathbf{r}_{1},\mathbf{r}_{2},\omega)=a^{*}(\mathbf{r}_{1},\omega)a(\mathbf{r}_{2},\omega)\frac{R_{2}J_{1}(2\pi\left|\mathbf{r}_{d}\right|R_{2})-R_{1}J_{1}(2\pi\left|\mathbf{r}_{d}\right|R_{1})}{\pi(R_{2}^{2}-R_{1}^{2})\left|\mathbf{r}_{d}\right|},$$

where *J*_1_ is Bessel function of the first kind. At the same argument, it becomes the spectral density

$$S(\mathbf{r},\omega)=W(\mathbf{r},\mathbf{r},\omega)=|a(\mathbf{r},\omega)|^{2}.$$

Therefore, the mutual coherence function of the incident field is

$$\Gamma_{ii}(\mathbf{r},\tau)=\int_{0}^{\infty} |a(\mathbf{r},\omega)|^{2}e^{-i\omega\tau}d\omega.$$

**Supplementary Note 2: Discussion about the contribution of the term** $\zeta(\mathbf{r})$

We will now thoroughly discuss the second term $\zeta(\mathbf{r})$ in Eq. . We will also compare this term to the Laplacian of the correlation function $\Re[\Gamma_{is}(\mathbf{r},\mathbf{r},-\pi/\left\langle\omega\right\rangle)]$, as well as the final RI distribution with and without the term $\zeta(\mathbf{r})$ in the reconstruction. Let us define the ratio

$$\eta(\mathbf{r})=\frac{2\Re\left[\int_{0}^{\infty} \langle\nabla U_{i}^{*}(\mathbf{r},\omega)\cdot\nabla U_{s}(\mathbf{r},\omega)\rangle e^{i\omega\pi/\left\langle\omega\right\rangle}d\omega\right]}{\nabla^{2}\Re[\Gamma_{is}(\mathbf{r},\mathbf{r},-\pi/\left\langle\omega\right\rangle)]}.$$

From four frames in SLIM, we can reconstruct the phase between the incident and scattered fields. The intensity of the incident and scatted fields can also be obtained from the phase-shifting measurements. Thus, we are able to calculate the dot product between the gradient of the incident field and the gradient of the scattered field. Based on our experimental data, $\zeta(\mathbf{r})$ contains no visible object structure. The Laplacian of the $\Re[\Gamma_{is}(\mathbf{r},\mathbf{r},-\pi/\left\langle\omega\right\rangle)]$ is also calculated from the phase-shifting measurements as illustrated in Supplemental note 1. It contains the edge information of the object. The ratio $\eta(\mathbf{r})$ is calculated and shown in Fig. S1 for three different samples. We applied the median filter to remove the isolated pixels. It can be seen that no object information is presented in the first row of Fig. S1. Most pixel values are close to zero. The inset in Fig. S1(c) represents the histogram of the values of $\eta(\mathbf{r})$. The second row in Fig. S1 illustrates the RI distributions for three different samples reconstructed with $\zeta(\mathbf{r})$in Eq. (11b). The third row in Fig. S1 presents the RI maps reconstructed without $\zeta(\mathbf{r})$. We can see that there is no difference between the two RI maps. Therefore, we conclude that the second term in Eq. does not play an important role in the RI reconstruction.


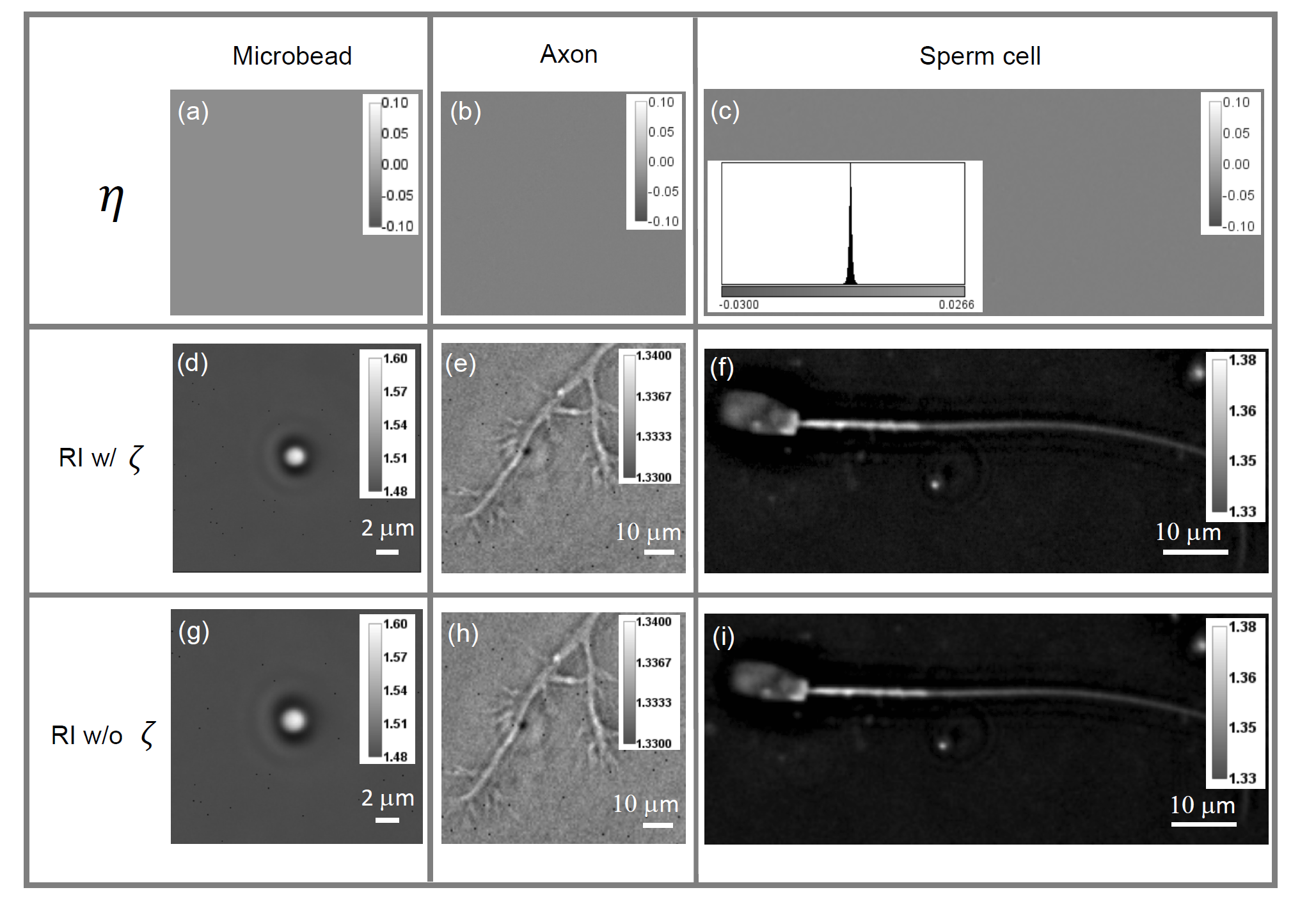


*Fig. S1. Comparison of the first two terms in Eq. . (a)-(c) $\eta(\mathbf{r})$for a microbead, axon, and sperm cell, as indicated. (d)-(f) The RI reconstructed with $\zeta(\mathbf{r})$. (g)-(i) The RI reconstructed without $\zeta(\mathbf{r})$.*

1. Mandel, L. and E. Wolf, *Optical Coherence and Quantum Optics*. 1995, Cambridge: Cambridge University Press.

2. Chen, X. and O. Korotkova, *Phase structuring of 2D complex coherence states.* Opt Lett, 2019. **44**(10): p. 2470-2473.
